# Effectiveness of clopidogrel vs. ticagrelor based on the ABCD-GENE score in acute coronary syndrome patients following percutaneous coronary intervention

**DOI:** 10.3389/fphar.2025.1606327

**Published:** 2025-06-11

**Authors:** Xiaotong Xu, Kun Na, Miaohan Qiu, Xueqing Yang, Zizhao Qi, Jing Li, Kai Xu, Xiaozeng Wang, Yi Li, Yaling Han

**Affiliations:** ^1^ State Key Laboratory of Frigid Zone Cardiovascular Disease, Cardiovascular Research Institute and Department of Cardiology, General Hospital of Northern Theater Command, Shenyang, China; ^2^ School of Life Science and Biochemistry, Shenyang Pharmaceutical University, Shenyang, China; ^3^ Department of Cardiology, the Second Affiliated Hospital of Harbin Medical University, Harbin, China

**Keywords:** CYP2C19 polymorphism, ABCD-GENE score, clopidogrel, ticagrelor, percutaneous coronary intervention

## Abstract

**Aim:**

This study employs the ABCD-GENE score (age, body mass index, chronic kidney disease, diabetes, and CYP2C19 variants) to compare the effectiveness and safety of clopidogrel versus ticagrelor-based DAPT in ACS patients post-PCI.

**Methods:**

A total of consecutive 21,705 ACS patients who underwent PCI between March 2016 and March 2023 and survived at discharge were included. The primary outcome was a composite of ischemic and bleeding events within 12 months, including cardiac death, myocardial infarction, ischemic stroke, and BARC types 3 or 5 bleeding. Propensity score matching was performed to balance baseline characteristics between clopidogrel and ticagrelor-based DAPT.

**Results:**

In the ABCD-GENE score <10 group, (4,748 matched pairs), ticagrelor increased BARC 3 or 5 bleeding (1.9% vs. 1.1%; HR: 1.52; 95% CI, 1.18–1.96; P = 0.0018), with no difference in the primary outcome (3.0% vs 3.5%; HR: 1.17; 95% CI: 0.94–1.46; P = 0.17) or ischemic events (2.0% vs 1.6%; HR: 0.82; 95% CI: 0.60–1.10; P = 0.19), compared with clopidogrel. In the ABCD-GENE score ≥10 group (1,231 matched pairs), ticagrelor significantly reduced the primary outcome (4.1% vs 6.0%; HR: 0.67; 95% CI: 0.47–0.96; P = 0.0272), driven by reduced rates of ischemic events (2.2% vs 4.5%; HR: 0.57; 95% CI: 0.38–0.85; P = 0.0015), without an increase in BARC 3 or 5 bleeding (1.9% vs. 1.7%; HR: 1.08; 95% CI, 0.60–1.96; P = 0.79), compared with clopidogrel.

**Conclusion:**

The ABCD-GENE score showed good predictive accuracy for a composite of ischemic and bleeding events and could identify patients likely to benefit from the ticagrelor-based antiplatelet strategy.

## Graphical Abstract

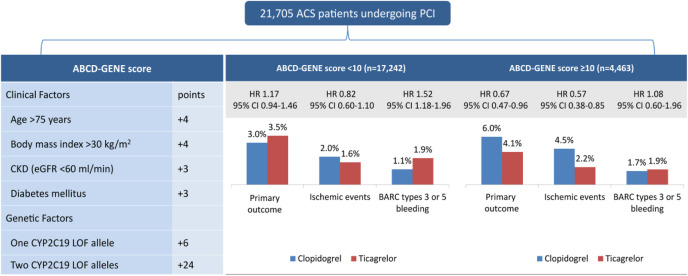

## Introduction

Dual antiplatelet therapy (DAPT) constitutes a cornerstone in the management of patients following percutaneous coronary intervention (PCI), playing an indispensable role in reducing atherothrombotic events (Nazir et al., 2021; Thomas et al., 2022; Byrne et al., 2023). However, optimizing the efficacy and safety of DAPT necessitates a precise balance between the complex dialectical relationship of ischemic and bleeding risks (Zhang et al., 2022). Within this clinical domain, the significant interindividual variability in response to P2Y12 inhibitors presents a challenge that demands urgent resolution. This heterogeneity is influenced not only by clinical factors (Na et al., 2022; Qiu et al., 2023; Tobita et al., 2023) but also governed by genetic determinants (Nguyen et al., 2022), with polymorphisms in the cytochrome P450 2C19 (CYP2C19) gene (Tazaki et al., 2012) playing a pivotal role in clopidogrel metabolism (Hu et al., 2024; Pereira et al., 2024). This multifaceted response heterogeneity underscores the imperative need for implementing individualized antiplatelet strategies in acute coronary syndrome (ACS) patients, strategies that comprehensively consider the intricate interplay between clinical parameters and genetic characteristics.

The ABCD-GENE score, which integrates age, body mass index (BMI), chronic kidney disease (CKD), diabetes, and CYP2C19 genetic variants, was developed as a pragmatic clinical tool to identify patients at elevated risk for high on-treatment platelet reactivity (HPR) when receiving clopidogrel therapy (Angiolillo et al., 2020; Bittl, 2020). Notably, East Asian populations exhibit significantly higher carrier rates of CYP2C19 loss-of-function alleles compared to Western cohorts, a population-specific genetic distribution pattern that confers profound significance to genetically-guided antiplatelet therapy in our clinical practice (Galli et al., 2024a; Pereira et al., 2024). Although several studies have validated the predictive value of the ABCD-GENE score for clopidogrel response (Capodanno et al., 2022; Jin et al., 2022; Thomas et al., 2022; Tan et al., 2023; AlSaeed et al., 2024; Thomas et al., 2024), the comparative effectiveness of clopidogrel versus ticagrelor stratified by ABCD-GENE score has not been systematically and comprehensively investigated in ACS patients undergoing PCI.

Despite substantial advancements in antiplatelet therapy, a fundamental challenge persists in contemporary cardiovascular medicine: transitioning from standardized treatment protocols to precision-based strategies tailored to individual risk profiles. The ABCD-GENE score represents a promising framework for this paradigm shift through its integration of clinical and genetic determinants. However, robust empirical evidence supporting its utility in guiding specific P2Y12 inhibitor selection remains notably absent from the literature. This study addresses this critical knowledge gap by systematically evaluating how the ABCD-GENE score influences clinical outcomes in ACS patients treated with either clopidogrel or ticagrelor following PCI. Through this multidimensional risk assessment approach, we aim not only to generate evidence for therapeutic selection algorithms but also to establish a foundational framework for implementing precision cardiovascular medicine in clinical practice—potentially transforming antiplatelet therapy from empirically-driven approaches toward genetically-informed individualized care.

## Methods

### Data sources and population

This study was a retrospective analysis of a single-center, all-comer, prospective, real-world PCI registry in the General Hospital of Northern Theater Command (Na et al., 2022; Qiu et al., 2023). From March 2016 to March 2023, consecutive ACS patients receiving clopidogrel- or ticagrelor-based DAPT following PCI and survived at discharge were included. Exclusion criteria included: (1) incomplete data for the ABCD-GENE score calculated; (2) switch between P2Y12 inhibitors during hospitalization. It should be noted that Cangrelor, an intravenous P2Y12 inhibitor, was not utilized in this study due to its limited availability in China during the study period. The study was approved by ethics board of General Hospital of Northern Theater Command (Ethics No: Y (2025) 134 and requirement for written consent was waived. The study complies with the provisions of the Declaration of Helsinki. Data were collected using a standard web-based platform (CV-NET system, Crealife Technology, Beijing, China).

### CYP2C19 genotyping assessment

CYP2C19 genotyping was performed by collecting 3 mL of fasting venous blood from patients in the early morning (using EDTA Na as anticoagulant). DNA was extracted using the nucleic acid extraction and CYP2C19 genotyping kit provided by Shanghai Bai’ao Technology Co., Ltd. Genomic DNA was amplified by polymerase chain reaction (PCR), followed by hybridization and colorimetric detection. The data were analyzed using the Bai’ao Gene Chip imaging software for image scanning and analysis. This process identified the major CYP2C19 allelic variants, including *1 (wild-type, normal function), *2 and *3 (loss-of-function alleles). The fast-metabolizer genotype (wild-type homozygous CYP2C19 *1/*1), intermediate metabolizer phenotypes (*1/*2, *1/*3), and poor metabolizer phenotypes (*2/*2, *2/*3, *3/*3) were determined. The turnaround time for genotyping results was approximately 24–48 h after blood collection, which enabled the treating physicians to make informed decisions about P2Y12 inhibitor selection before patient discharge.

### Risk assessment and secondary prevention medications

The ABCD-GENE score (Angiolillo et al., 2020) was calculated based on patient characteristics at the time of the undergoing PCI, which includes four clinical factors and one genetic factor. The clinical factors were age >75 years (4 points), BMI >30 kg/m^2^ (4 points), CKD, defined as estimated glomerular filtration rate <60 mL/min/1.73 m^2^) (3 points), and diabetes (3 points). The genetic factor was the presence of one CYP2C19 loss-of-function (LOF) allele (6 points), and two CYP2C19 LOF alleles (24 points). A cut-off score of ≥10 was identified as the optimal threshold for identifying an increased risk of developing high platelet reactivity (HPR) and adverse ischemia events, as demonstrated in the proof-of-concept study (Angiolillo et al., 2020).

The choice of P2Y12 receptor inhibitor was at the treating physician’s discretion, with the recommendation of standard 12-month dual-antiplatelet therapy for all patients. Secondary prevention medications, including statins, angiotensin-converting enzyme inhibitors/angiotensin receptor blockers (ACEIs/ARBs), and beta-blockers, were recommended based on clinical judgment.

### Outcomes and Definitions

The primary outcome was a composite of ischemic and bleeding events at 12 months after discharge, including cardiac death, MI, stroke, and/or Bleeding Academic Research Consortium (BARC) types 3 or 5 bleeding events. In this study, cardiac death was defined as death resulting from acute myocardial infarction, heart failure, arrhythmia, or other cardiac-related complications. Secondary endpoints included 12-month all-cause death, ischemic events (a composite of cardiac death, MI, and/or stroke), BARC types 2, 3 or 5 bleeding, and BARC types 3 or 5 bleeding. All patients were followed-up by telephone or email at 3, 6, 9, and 12 months.

### Statistical analysis

Continuous variables were expressed as mean with standard deviation, and the differences between groups were assessed by the Student’s t-test. Categorical variables were expressed as frequencies and percentages, and between-group differences were compared using either the Pearson χ^2^ test or the Fisher exact test. Time-to-event data with estimated event rates, which were measured with the Kaplan-Meier method were compared using the log-rank test. Cox proportional hazards models were used to estimate the hazard ratio (HR) and 95% confidence interval (CI) for each outcome among the groups. To minimize the bias of confounders on outcomes, propensity score matching analysis was performed in patients within ABCD-GENE <10 and ABCD-GENE ≥10, respectively. The propensity score used the nearest matching neighbor that included age, sex, BMI, hypertension, diabetes, previous MI, previous stroke, previous PCI, smoking status, presentation, estimated glomerular filtration rate (eGFR), anemia, procedure information (arterial access, coronary arteries treated, number of stents, total length of stents, average stent diameters), and medical treatment at discharge. Statistical analysis was performed using SAS software version 9.4 (SAS Institute, Cary, NC, USA). A two-sided P value less than 0.05 indicated statistical significance.

## Results

### Population characteristics

A total of consecutive 21,705 ACS patients undergoing PCI with a complete ABCD-GENE score were included in the present analysis. During the 12-month observation period, a follow-up rate of 99.7% was achieved. Of these, 17,242 (79.43%) had an ABCD-GENE score <10, and 4,463 (20.56%) had a score ≥10. Clinical characteristics significantly differed between groups, with a higher prevalence of ABCD-GENE score components in the ≥10 group, as expected. Additionally, the high score group had a greater proportion of women and more frequently presented with hypertension and a history of previous stroke. In contrast, current smokers were more common among patients with low ABCD-GENE scores. Despite these differences in clinical profiles, procedural characteristics, such as trans-radial access, the number of stents, total length of stents, and average stent diameters, were similar between the two groups (Supplementary Table S1). Notably, the distribution of CYP2C19 metabolizer phenotypes varied significantly between the two groups (Supplementary Table S2). Notably, CYP2C19 loss-of-function allele carriers constituted 47.5% (8,194/17,242) of patients in the <10 group and 98.0% (4,372/4,463) of patients in the ≥10 group (Supplementary Table S2).

### Antiplatelet therapy during follow-up

During the 12-month follow-up period, adherence to dual antiplatelet therapy remained high in both treatment groups, with 91.8% of patients in the clopidogrel group and 93.5% in the ticagrelor group maintaining DAPT. P2Y12 inhibitor monotherapy was observed in 5.9% of the clopidogrel group and 4.9% of the ticagrelor group, while aspirin monotherapy was used in 1.2% and 0.9%, respectively. Complete discontinuation of antiplatelet therapy was rare in both groups (1.0% in clopidogrel vs 0.8% in ticagrelor). Notably, P2Y12 inhibitor switching occurred more frequently among patients receiving ticagrelor compared to those receiving clopidogrel (5.5% vs 3.5%, respectively). Details of antiplatelet therapy regimens during follow-up are presented in Supplementary Table S3.

### Clinical outcomes stratified by ABCD-GENE score

In the overall population, the primary outcomes were more common among patients with ABCD-GENE scores ≥10 compared with those with scores <10 (5.2% vs. 3.6%; HR, 1.46; 95% CI, 1.25–1.0001; P < 0.0001), driven by higher rates of cardiac death (2.0% vs. 1.0%; HR,1.93; 95% CI, 1.50–2.49; P < 0.0001) and BARC types 3 or 5 bleeding (2.0% vs. 1.5%; HR, 1.34; 95% CI, 1.05–1.71; P = 0.02). Additionally, patients with a score ≥10 experienced a significantly higher incidence of all-cause death compared to those with a score <10 (2.8% vs. 1.4%; HR, 2.02; 95% CI, 1.63–2.51; P < 0.0001). In contrast, no significant differences were observed between the two groups in the rates of BARC types 2, 3 or 5 bleeding (7.2% vs 6.7%; HR, 1.09; 95% CI, 0.97–1.24; P = 0.16), non-fatal MI (0.7% vs 0.6%; HR, 1.25; 95% CI, 0.83–1.87; P = 0.28), or ischemic stroke (1.0% vs 0.8%; HR, 1.25; 95% CI, 0.89–1.76; P = 0.20) (Table 1). Kaplan–Meier curves are shown in Supplementary Figure S1.

**TABLE 1:** Clinical outcomes between ABCD-GENE scores ≥10 and those with scores <10.

	ABCD-gene score		HR (95%CI)	P value
	ABCD-gene score <10 (N = 17,242)	ABCD-gene score ≥10 (N = 4463)		
Primary outcome	616 (3.6%)	230 (5.2%)	1.46 (1.25–1.70)	<0.0001
Ischemic events	380 (2.2%)	148 (3.3%)	1.52 (1.26–1.84)	<0.0001
Cardiac death	179 (1.0%)	89 (2.0%)	1.93 (1.50–2.49)	<0.0001
MI	97 (0.6%)	31 (0.7%)	1.25 (0.83–1.87)	0.2841
Stroke	134 (0.8%)	43 (1.0%)	1.25 (0.89–1.76)	0.2004
All-cause death	237 (1.4%)	123 (2.8%)	2.02 (1.63–2.51)	<0.0001
BARC types 2,3 or 5 bleeding	1156 (6.7%)	323 (7.2%)	1.09 (0.97–1.24)	0.1611
BARC types 3 or 5 bleeding	259 (1.5%)	89 (2.0%)	1.34 (1.05–1.71)	0.0166

Abbreviation: BARC, bleeding academic research consortium; CI, confidence interval; HR, hazard ratio; MI, myocardial infarction.

The primary outcome was defined as the composite of cardiac death, MI, stroke, or BARC, types 3 or 5 bleeding.

### Ticagrelor vs. clopidogrel among patients with ABCD-GENE scores <10

Among patients in the low-score stratified cohort (ABCD-GENE score <10), ticagrelor-treated patients were generally younger, had a higher average BMI, and were more likely to be male compared to clopidogrel-treated patients. They also had a higher prevalence of previous MI and previous stroke. Conversely, they were less likely to have hypertension, CKD, or anemia. At discharge, ticagrelor-treated patients were more frequently prescribed secondary prevention medications, including aspirin, β-blockers, statins, and ACEI/ARBs, as detailed in Supplementary Table S4.

After propensity score matching (4,748 matched pairs), baseline characteristics were well balanced between ticagrelor and clopidogrel groups (Table 2). Analysis revealed no significant differences in the primary outcome (3.5% vs 3.0%; HR, 1.17; 95% CI, 0.94–1.46; P = 0.1704), ischemic events (1.6% vs 2.0%; HR, 0.82; 95% CI, 0.60–1.10; P = 0.1851), or all-cause death (0.9% vs 1.2%; HR, 0.71; 95% CI, 0.48–1.05; P = 0.09) between the two groups. However, clopidogrel treatment was associated with significantly lower rates of BARC types 2, 3 or 5 bleeding (9.2% vs 5.4%; HR, 1.73; 95% CI, 1.48–2.01; P < 0.0001) and BARC types 3 or 5 bleeding (1.9% vs 1.1%; HR, 1.71; 95% CI, 1.22–2.39; P = 0.0018) compared to ticagrelor (Table 3). These results were consistent with outcomes in the unmatched cohort (Supplementary Table S5).

**TABLE 2:** Baseline and procedural characteristics based on P2Y12 treatment and ABCD-GENE score after propensity score matching.

After match	ABCD-GENE score <10 (N = 17,242)				ABCD-GENE score ≥10 (N = 4463)			
	Clopidogrel (N = 4748)	Ticagrelor (N = 4748)	Standardized mean difference	P value	Clopidogrel (N = 1231)	Ticagrelor (N = 1231)	Standardized mean difference	P value
Age	57.8 ± 9.8	57.9 ± 9.2	0.0144	0.4819	58.9 ± 11.1	58.9 ± 9.8	−0.0065	0.8729
Male	3861 (81.3%)	3837 (80.8%)	−0.0129	0.5296	931 (75.6%)	948 (77.0%)	0.0325	0.4203
Body mass index, kg/m^2^ ^a^	25.1 ± 3.1	25.2 ± 3.0	0.0249	0.2244	26.8 ± 4.6	26.8 ± 4.2	0.0037	0.9268
Medical history								
Hypertension	2764 (58.2%)	2761 (58.2%)	−0.0013	0.9502	783 (63.6%)	800 (65.1%)	0.031	0.4414
Diabetes	1455 (30.6%)	1463 (30.8%)	0.0037	0.8588	456 (37.0%)	423 (34.4%)	−0.056	0.1651
Previous MI	706 (14.9%)	701 (14.8%)	−0.003	0.8852	169 (13.8%)	178 (14.5%)	0.0204	0.6136
Previous stroke	430 (9.1%)	435 (9.2%)	0.0037	0.8585	159 (12.9%)	151 (12.3%)	−0.0196	0.6269
Previous PCI	906 (19.1%)	921 (19.4%)	0.008	0.6962	239 (19.4%)	238 (19.3%)	−0.0029	0.9436
History of bleeding	29 (0.6%)	24 (0.5%)	−0.0141	0.4900	7 (0.6%)	15 (1.2%)	0.0691	0.0900
Smoking			0.019	0.6502			0.0212	0.8713
Never	1807 (38.1%)	1851 (39.0%)			519 (42.3%)	529 (43.0%)		
Active	2263 (47.7%)	2229 (46.9%)			545 (44.4%)	533 (43.3%)		
Former	678 (14.3%)	668 (14.1%)			164 (13.4%)	168 (13.7%)		
Presentation			0.0217	0.5706			0.0402	0.6077
UA	2859 (60.2%)	2821 (59.4%)			770 (62.6%)	746 (60.6%)		
NSTEMI	813 (17.1%)	851 (17.9%)			218 (17.7%)	228 (18.5%)		
STEMI	1076 (22.7%)	1076 (22.7%)			243 (19.7%)	257 (20.9%)		
eGFR, ml/min/1.73m^2^ ^b^	96.1 ± 25.3	96.4 ± 23.0	0.0089	0.6642	90.1 ± 28.4	91.0 ± 25.0	0.0341	0.3982
Anemia^c^	1525 (32.1%)	1562 (32.9%)	0.0166	0.4176	442 (36.0%)	448 (36.5%)	0.0114	0.7777
LVEF	57.4 ± 8.8	57.6 ± 8.4	0.0241	0.3018	57.3 ± 8.8	57.1 ± 8.9	−0.0206	0.6529
CYP2C19 genotype			−0.1059	<0.0001			0.2125	<0.0001
0 LOF alleles (NM, RM, UM)	2541 (53.5%)	2290 (48.2%)			15 (1.2%)	7 (0.6%)		
1 LOF allele (IM)	2207 (46.5%)	2458 (51.8%)			424 (34.4%)	314 (25.5%)		
2 LOF alleles (PM)	0 (0.0%)	0 (0.0%)			792 (64.3%)	910 (73.9%)		
Procedure information								
Transradial access	4484 (94.4%)	4472 (94.2%)	−0.0109	0.5949	1164 (94.6%)	1168 (94.9%)	0.0145	0.7185
Coronary arteries treated								
LM	306 (6.4%)	316 (6.7%)	0.0085	0.6783	53 (4.3%)	57 (4.6%)	0.0157	0.6964
LAD	2595 (54.7%)	2577 (54.3%)	−0.0076	0.7107	649 (52.7%)	666 (54.1%)	0.0277	0.4922
LCX	1264 (26.6%)	1264 (26.6%)	0	>0.9999	355 (28.8%)	345 (28.0%)	−0.018	0.655
RCA	1725 (36.3%)	1731 (36.5%)	0.0026	0.8982	461 (37.4%)	463 (37.6%)	0.0034	0.9337
Number of stents	1.7 ± 1.0	1.7 ± 1.0	0.0051	0.8021	1.6 ± 0.9	1.7 ± 1.0	0.0432	0.2837
Total length of stents, mm	45.0 ± 28.9	45.2 ± 28.4	0.0084	0.6835	42.7 ± 27.5	44.0 ± 28.2	0.0457	0.2569
Average stent diameters, mm	2.9 ± 0.8	2.9 ± 0.8	−0.01	0.6246	2.8 ± 0.8	2.8 ± 0.8	0.0012	0.9765
Medical treatment at discharge								
Aspirin	4731 (99.6%)	4729 (99.6%)	−0.0069	0.7384	1228 (99.8%)	1225 (99.5%)	−0.0404	0.3164
Statins	4416 (93.0%)	4429 (93.3%)	0.0108	0.5975	1143 (92.9%)	1157 (94.0%)	0.0459	0.2551
ACEI/ARB	2619 (55.2%)	2601 (54.8%)	−0.0076	0.7104	662 (53.8%)	695 (56.5%)	0.0539	0.1812
βblockers	3011 (63.4%)	3037 (64.0%)	0.0114	0.579	760 (61.7%)	777 (63.1%)	0.0285	0.4793
PPI	1555 (32.8%)	1586 (33.4%)	0.0139	0.4989	375 (30.5%)	381 (31.0%)	0.0106	0.7932

Abbreviations: ACEI, angiotensin-converting enzyme inhibitor; ARB, angiotensin II, receptor blocker; eGFR, estimated glomerular filtration rate; LVEF, left ventricular ejection fraction; LM, left main coronary artery; LAD, left anterior decending branch; LCX, left circumflex branch; MI, myocardial infarction; NSTEMI, Non-ST -segment-elevation myocardial infarction; PCI, percutaneous coronary intervention; PPIs, proton pump inhibitors; RCA, right coronary artery; STEMI, ST-segment-elevation myocardial infarction; UA, unstable angina.

^a^ Calculated as weight in kilograms divided by height in meters squared.

^b^ Calculated as milliliters per minute per 1.73 square meters.

^c^ Anemia was defined as hemoglobin (less than 130 g/L for male patients and less than 120 g/L for female patients.

**TABLE 3:** Clinical outcomes for patients based on P2Y12 treatment and ABCD-GENE score after propensity score matching.

	ABCD-GENE score <10 (N = 17,242)				ABCD-GENE score ≥10 (N = 4463)			
	Clopidogrel (N = 4748)	Ticagrelor (N = 4748)	HR (95%CI)	P value	Clopidogrel (N = 1231)	Ticagrelor (N = 1231)	HR (95%CI)	P value
Primary outcome	143 (3.0%)	167 (3.5%)	1.17 (0.94–1.46)	0.1704	74 (6.0%)	50 (4.1%)	0.67 (0.47–0.96)	0.0272
Ischemic events	94 (2.0%)	77 (1.6%)	0.82 (0.60–1.10)	0.1851	56 (4.5%)	27 (2.2%)	0.48 (0.30–0.75)	0.0015
Cardiac death	46 (1.0%)	36 (0.8%)	0.78 (0.51–1.21)	0.2678	37 (3.0%)	12 (1.0%)	0.32 (0.17–0.62)	0.0006
MI	18 (0.4%)	25 (0.5%)	1.39 (0.76–2.54)	0.2913	9 (0.7%)	8 (0.6%)	0.88 (0.34–2.28)	0.7886
Stroke	37 (0.8%)	23 (0.5%)	0.62 (0.37–1.04)	0.0703	16 (1.3%)	8 (0.6%)	0.49 (0.21–1.15)	0.1006
All-cause death	59 (1.2%)	42 (0.9%)	0.71 (0.48–1.05)	0.09	46 (3.7%)	15 (1.2%)	0.32 (0.18–0.58)	0.0001
BARC types 2,3 or 5 bleeding	258 (5.4%)	435 (9.2%)	1.73 (1.48–2.01)	<0.0001	70 (5.7%)	98 (8.0%)	1.41 (1.04–1.91)	0.029
BARC types 3 or 5 bleeding	54 (1.1%)	92 (1.9%)	1.71 (1.22–2.39)	0.0018	21 (1.7%)	23 (1.9%)	1.08 (0.60–1.96)	0.79

Abbreviation: BARC, bleeding academic research consortium; CI, confidence interval; HR, hazard ratio; MI, myocardial infarction. The primary outcome was defined as the composite of cardiac death, MI, stroke, or BARC, types 3 or 5 bleeding.

### Ticagrelor vs. clopidogrel among patients with ABCD-GENE scores ≥10

In the high-score stratified cohort (ABCD-GENE score ≥10), differences in age, sex, smoking status, PCI indication, medical history, and concomitant medications were also observed between the clopidogrel and ticagrelor therapy groups. In general, ticagrelor-treated patients tended to be younger, had fewer comorbidities, and were more likely to receive secondary prevention medications (Supplementary Table S4).

Propensity score matching (1,231 matched pairs) minimized imbalances in baseline variables for patients with an ABCD-GENE score ≥10 (Table 2). In the matched cohort, ticagrelor use, compared with clopidogrel, was associated with a significantly lower risk of the primary outcome at 12 months (4.1% vs. 6.0%; HR, 0.67; 95% CI, 0.47–0.96; P = 0.0272), primarily driven by a significantly lower risk of cardiac death (1.0% vs. 3.0%; HR, 0.32; 95% CI, 0.17–0.62; P = 0.0006). Additionally, ticagrelor-treated patients experienced a significantly lower rate of all-cause mortality compared to those treated with clopidogrel (1.2% vs. 3.7%; HR, 0.32; 95% CI, 0.18–0.58; P = 0.0001). While no significant differences were observed between the two groups in terms of BARC types 3 or 5 bleeding events (1.9% vs. 1.7%; HR, 1.08; 95% CI, 0.60–1.96; P = 0.79), the incidence of BARC types 2, 3 or 5 bleeding was higher in the ticagrelor group (8.0% vs. 5.7%; HR, 1.41; 95% CI, 1.04–1.91; P = 0.029) (Table 3).

**FIGURE 1:**
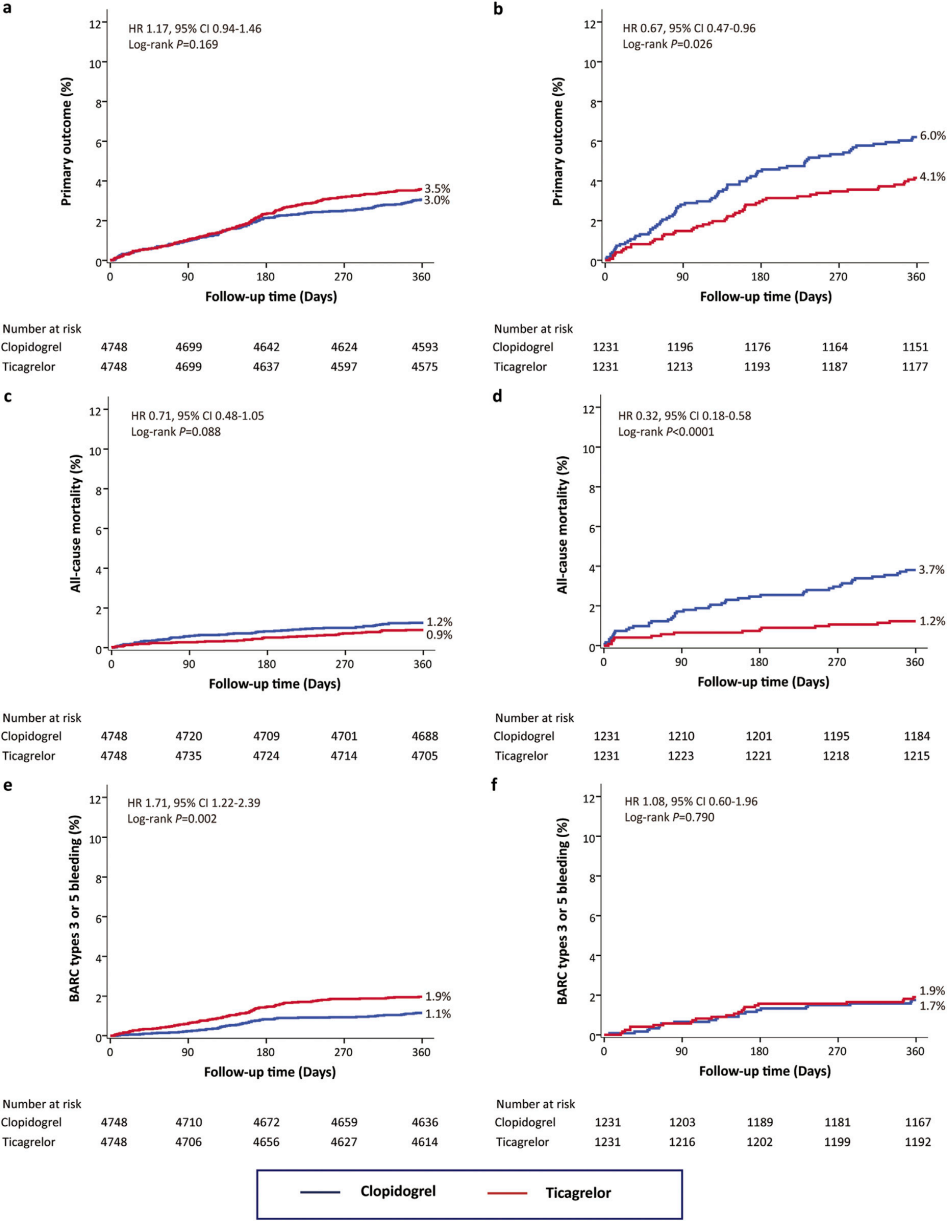
The 12-month Kaplan–Meier cumulative event curves for clinical outcomes based on P2Y12 treatment and ABCD-GENE score after propensity score matching. Curved depict the incidence of the primary outcome **(a, b)**, all-cause death **(c, d)**, and BARC types 3 or 5 bleeding **(e, f)**. Curves are provided both ABCD-GENE score <10 **(a, c, e)** and ABCD-GENE score ≥10 **(b, d, f)**. The primary outcome was defined as the composite of myocardial infarction (MI), stroke, and/or BARC types 3 or 5 bleeding events. BARC, bleeding academic research consortium.

Kaplan-Meier curves for clinical outcomes were depicted for patients with an ABCD-GENE score ≥10 and <10, stratified by P2Y12 prescription, in Figure 1 and Supplementary Figure S1.

## Discussion

This large-scale retrospective analysis of 21,705 ACS patients undergoing PCI systematically evaluated the comparative efficacy and safety profiles of clopidogrel versus ticagrelor stratified by ABCD-GENE scores. The findings substantiate the discriminative capacity of the ABCD-GENE score in risk stratification based on integrated clinical and genetic parameters, thereby facilitating individualized antiplatelet therapy selection aligned with patient-specific risk profiles.

Long-term antithrombotic strategies post PCI in patients with ACS have always played a very important role in their prognostic management, and precise and rational antithrombotic strategies can significantly improve the long-term prognosis of patients. Previous studies have demonstrated that guiding the selection of P2Y12 receptor inhibitors through platelet function and genetic monitoring is feasible (Galli et al., 2021; Galli et al., 2022; Angiolillo et al., 2024). The ABCD-GENE score integrates four clinical factors affecting the activity of participating platelets and the CYP2C19 genotype, enhancing the identification of patients with HPR. (Angiolillo et al., 2020). Prior studies have shown that guiding the selection of P2Y12 receptor inhibitors through platelet function and genetic monitoring is feasible. CYP2C19 loss-of-function alleles significantly affect clopidogrel efficacy (Alkattan et al., 2021; Nguyen et al., 2022), compared with clopidogrel, ticagrelor or prasugrel more effectively reduce platelet reactivity and the occurrence of ischemic events in ACS patients carrying CYP2C19 loss-of-function alleles (Galli et al., 2024b; Galli et al., 2024c), while these genetic insights were not fully utilized in earlier risk models. Furthermore, the higher prevalence of CYP2C19 LOF alleles in East Asian populations (Yasuda et al., 2019; Alkattan et al., 2021; Pereira et al., 2024) underscores the importance of incorporating genotyping into risk assessments (Saito et al., 2022; Goto et al., 2023). Our research reinforces the predictive validity of the ABCD-GENE score across diverse populations, highlighting both the necessity and feasibility of individualized therapy. Patients with higher scores often present with multiple risk factors that can affect both thrombotic risk and drug metabolism. The combination of clinical factors (age, BMI, CKD and diabetes) with CYP2C19 genetic variants likely captures both the pharmacokinetic and pharmacodynamic aspects of antiplatelet response, advancing the application of precision medicine in cardiovascular disease management.

Although contemporary guidelines recommend potent P2Y12 inhibition with ticagrelor or prasugrel as the first-line choice for patients with ACS undergoing PCI (Byrne et al., 2023; Rao et al., 2025), clopidogrel remains the most commonly used P2Y12 inhibitor and represents the agent of choice for most East Asian patients (Adamiak-Giera et al., 2021; Peng et al., 2023). A comprehensive assessment of clinical and genetic factors is essential to guide evidence-based appropriate antiplatelet therapy decision-making. Our study suggests that in patients with ABCD-GENE scores <10, clopidogrel shows similar efficacy to ticagrelor in preventing the primary outcome while offering a superior safety profile with significantly lower bleeding rates. These results underscore the practicality of continuing clopidogrel in low-risk populations, particularly given its affordability and broader applicability in real-world settings. Conversely, in patients with ABCD-GENE scores ≥10, ticagrelor was associated with a significant reduction in the risk of all-cause death and primary outcome events despite a slight increase in minor bleeding events. This finding is in contrast to the results of a previous study conducted on a US population (Thomas et al., 2024), which revealed no significant difference in the risk of ischemic events between clopidogrel and alternative P2Y12 Inhibitors (prasugrel or ticagrelor), even among patients with ABCD-GENE scores ≥10. The potential factors contributing to these observed differences are likely to be multifaceted. Firstly, substantial differences in sample size and baseline characteristics were observed: the present study included a larger population with scores ≥10 (N = 4,463) and a higher proportion of genetically tested poor metabolizer (PM) (59.9% vs 10.1%) compared with previous studies (N = 1,135), and the significantly lower metabolising capacity of PM for clopidogrel resulted in a more pronounced advantage of ticagrelor in this population. In addition, our study utilized PSM to balance baseline characteristics (e.g., age, sex, diabetes), thereby minimizing the impact of 'healthy user bias'. The differential response to antiplatelet therapy based on ABCD-GENE scores supports the biological premise outlined in previous studies regarding CYP2C19 genetic variations (Alkattan et al., 2021; Nguyen et al., 2022) and clinical risk factors (Backus et al., 2011; Al-Daydamony and Farag, 2016).

The integration of both genetic and clinical factors in the ABCD-GENE score represents a paradigm shift in personalized antiplatelet therapy, offering a more comprehensive and holistic approach to risk assessment. By considering the intricate interplay between an individual’s unique genetic profile and their clinical characteristics, the ABCD-GENE score enables physicians to move beyond a one-size-fits-all approach and tailor treatment strategies to each patient’s specific needs.

Notably, despite the theoretical advantages of individualized antiplatelet therapy strategies, significant challenges remain in clinical implementation. Observational data revealed differential P2Y12 inhibitor switching patterns between ticagrelor and clopidogrel cohorts (5.5% versus 3.5%), potentially reflecting real-world variations in pharmacological tolerability. Ticagrelor-associated adverse events (particularly dyspneic manifestations) coupled with elevated treatment expenditures may contribute to increased therapy modification rates, consistent with other investigational findings. Although the study population demonstrated high 12-month adherence rates, medication persistence remains a critical determinant of clinical outcomes, with contemporary literature indicating only 54.8% of patients maintain consistent P2Y12 inhibitor therapy regimens (Turgeon et al., 2022). Premature DAPT discontinuation significantly augments adverse event probability, emphasizing the critical importance of comprehensive adherence pattern monitoring beyond simplistic compliance metrics.

The uniform 12-month DAPT protocol implemented in this study, while methodologically necessary for analytical consistency, may not optimize therapeutic outcomes across heterogeneous patient subpopulations. For patients with ABCD-GENE scores ≥10, maintenance of potent P2Y12 inhibition appears essential due to elevated ischemic risk profiles, whereas patients with scores <10, particularly those exhibiting high bleeding risk characteristics, might derive greater benefit from abbreviated DAPT duration or de-escalation strategies. This observation suggests that ABCD-GENE score utility potentially extends beyond initial pharmacological selection, informing comprehensive treatment algorithms including therapy duration and intensity modifications. Future investigations should evaluate how the ABCD-GENE score might guide longitudinal personalized antiplatelet strategies throughout the treatment continuum. Such integrated precision approaches could optimize individual benefit-risk profiles for post-PCI patients, representing meaningful progression from population-based toward patient-centered antiplatelet therapeutic paradigms.

### Limitations

The present study has several important limitations. First, despite employing propensity score matching, our retrospective design contains unmeasured confounders that could influence outcomes. Second, while we documented P2Y12 inhibitor switching (3.5% in clopidogrel vs 5.5% in ticagrelor groups), we did not systematically track early discontinuation or de-escalation strategies, which could potentially affect clinical outcomes. Thirdly, our study did not include Cangrelor, an intravenous P2Y12 inhibitor that has shown promising results in the peri-PCI phase, particularly in patients who cannot take oral medications or require immediate platelet inhibition. Recent evidence has demonstrated the efficacy of Cangrelor in the peri-PCI phase (Pepe et al., 2022; Pepe et al., 2023). The unavailability of Cangrelor in our clinical setting during the study period limited our ability to compare all potential antiplatelet strategies, representing a gap in our comprehensive analysis of P2Y12 inhibitor efficacy. Fourthly, while international guidelines suggest shorter DAPT durations for high-risk patients, our study employed a uniform 12-month regimen to maintain methodological consistency. This approach, while eliminating treatment duration as a variable, limited our ability to evaluate individualized DAPT strategies. Fifthly, the single-center nature of this study may limit generalizability. Future multi-center investigations are needed to explore how the ABCD-GENE score might guide both P2Y12 inhibitor selection and optimal DAPT duration, particularly in high bleeding risk patients where shortened regimens might provide a more favorable risk-benefit profile. Finally, this study exclusively enrolled Asian patients; therefore, the results should be confirmed in other populations.

## Conclusion

In conclusion, ABCD-GENE score was a remarkable tool to evaluate risk of ischemic and bleeding events in ACS patients receiving DAPT following PCI. Furthermore, ABCD-GENE score could identify patients who would gain the most benefit from a specific DAPT strategy.

## Data Availability

The raw data supporting the conclusions of this article will be made available by the authors, without undue reservation.
