# Supplementary figures and images for: Effectiveness of clopidogrel vs. ticagrelor based on the ABCD-GENE score in acute coronary syndrome patients following percutaneous coronary intervention
Source: Front Pharmacol. 2025 Jun 11;16:1606327. doi: 10.3389/fphar.2025.1606327 (PMC12187568; doi:10.3389/fphar.2025.1606327)

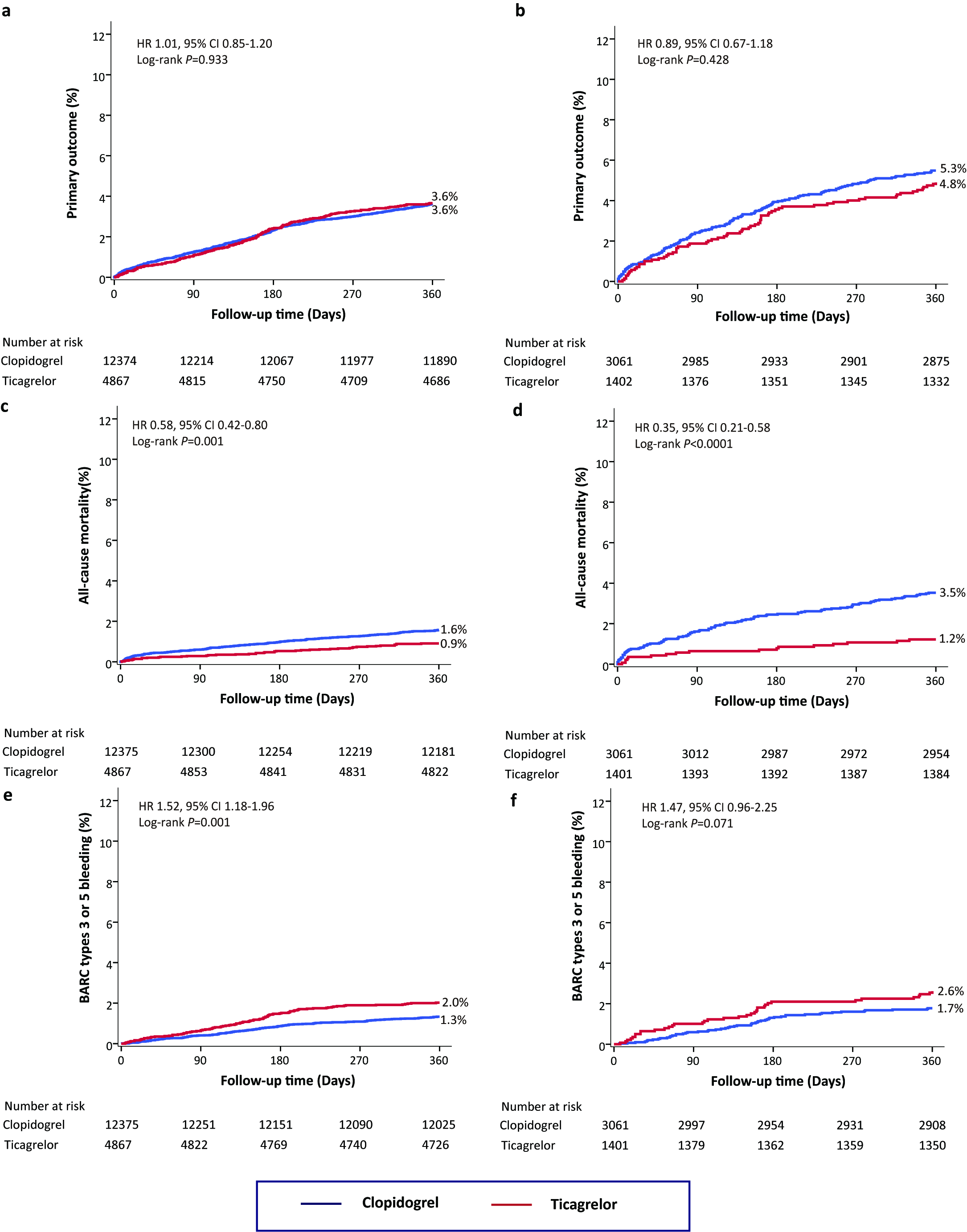

Supplement: Supplementary file 1 [file Image1.tif]
