# Supplementary material for: Effectiveness of clopidogrel vs. ticagrelor based on the ABCD-GENE score in acute coronary syndrome patients following percutaneous coronary intervention
Source: Front Pharmacol. 2025 Jun 11;16:1606327. doi: 10.3389/fphar.2025.1606327 (PMC12187568; doi:10.3389/fphar.2025.1606327)
Supplement: Supplementary file 2 [file DataSheet1.docx]

**Supplementary Table1. Baseline and Procedural Characteristics Stratified by ABCD-GENE Score**

|  | ABCD-gene score | |  |
| --- | --- | --- | --- |
|  | <10 (N=17242) | ≥10 (N=4463) | P value |
| Age | 60.4±9.6 | 63.6±12.3 | <0.0001 |
| Male | 12666 (73.5%) | 3074 (68.9%) | <0.0001 |
| Body mass index, kg/m^2 a^ | 25.0±3.0 | 26.2±4.4 | <0.0001 |
| Medical history |  |  |  |
| Hypertension | 10518 (61.0%) | 2983 (66.9%) | <0.0001 |
| Diabetes | 5423 (31.5%) | 1652 (37.0%) | <0.0001 |
| Previous MI | 2357 (13.7%) | 654 (14.7%) | 0.0934 |
| Previous stroke | 2205 (12.8%) | 653 (14.7%) | 0.0012 |
| Previous PCI | 3238 (18.8%) | 894 (20.0%) | 0.0569 |
| Smoking |  |  | <0.0001 |
| Never | 7727 (44.9%) | 2261 (50.7%) |  |
| Active | 7216 (41.9%) | 1647 (37.0%) |  |
| Former | 2272 (13.2%) | 549 (12.3%) |  |
| Presentation |  |  | <0.0001 |
| UA | 11386 (66.0%) | 2776 (62.2%) |  |
| NSTEMI | 2680 (15.5%) | 776 (17.4%) |  |
| STEMI | 3176 (18.4%) | 911 (20.4%) |  |
| eGFR, ml/min/1.73m^2 b^ | 94.2±24.1 | 85.4±28.7 | <0.0001 |
| Anemia ^c^ | 6058 (35.2%) | 1915 (43.0%) | <0.0001 |
| LVEF | 57.9±8.5 | 56.7±9.0 | <0.0001 |
| Procedure information |  |  |  |
| Transradial access | 16153 (93.9%) | 4162 (93.4%) | 0.1863 |
| Coronary arteries treated |  |  |  |
| LM | 902 (5.2%) | 236 (5.3%) | 0.88 |
| LAD | 9298 (53.9%) | 2363 (52.9%) | 0.2419 |
| LCX | 4522 (26.2%) | 1213 (27.2%) | 0.1984 |
| RCA | 6673 (38.7%) | 1747 (39.1%) | 0.5891 |
| Number of stents |  |  |  |
| Total length of stents, mm | 1.6±1.0 | 1.6±1.0 | 0.7116 |
| Average stent diameters, mm | 42.3±28.3 | 42.2±28.1 | 0.8623 |
| Medical treatment at discharge | 2.8±0.9 | 2.8±0.9 | 0.5496 |
| Aspirin | 16532 (95.9%) | 4225 (94.7%) | 0.0004 |
| P2Y12 inhibitor |  |  | <0.0001 |
| Clopidogrel | 12375 (71.8%) | 3061 (68.6%) |  |
| Ticagrelor | 4867 (28.2%) | 1402 (31.4%) |  |
| Statins | 15615 (90.6%) | 4023 (90.1%) | 0.3913 |
| ACEI/ARB | 8423 (48.9%) | 2277 (51.0%) | 0.0098 |
| βblockers | 9710 (56.3%) | 2564 (57.5%) | 0.1731 |
| PPIs | 5565 (32.3%) | 1386 (31.1%) | 0.1193 |

Abbreviations: ACEI, angiotensin-converting enzyme inhibitor; ARB, angiotensin II receptor blocker; CABG, coronary artery bypass grafting; eGFR, estimated glomerular filtration rate; LVEF, left ventricular ejection fraction; LM, left main coronary artery; LAD, left anterior decending branch; LCX, left circumflex branch; MI, myocardial infarction; NSTEMI, Non-ST -segment-elevation myocardial infarction; PCI, percutaneous coronary intervention; PPIs, proton pump inhibitors; RCA, right coronary artery; STEMI, ST-segment-elevation myocardial infarction; UA, unstable angina.

^a^ Calculated as weight in kilograms divided by height in meters squared.

^b^ Calculated as milliliters per minute per 1.73 square meters.

^c^ Anemia was defined as hemoglobin (less than 130 g/L for male patients and less than 120 g/L for female patients.

**Supplementary Table 2. Prevalence of the Clinical and Genetic Components of the ABCD-GENE Score for Patients Stratified by ABCD-GENE Score**

|  | ABCD-gene score | |  |
| --- | --- | --- | --- |
|  | <10 (N=17242) | ≥10 (N=4463) | P value |
| ABCD-GENE score |  |  |  |
| Age > 75 years | 605 (3.5%) | 980 (22.0%) | <0.0001 |
| BMI > 30 kg/m2 | 648 (3.8%) | 943 (21.1%) | <0.0001 |
| CKD | 1004 (5.8%) | 833 (18.7%) | <0.0001 |
| Diabetes | 5423 (31.5%) | 1652 (37.0%) | <0.0001 |
| CYP2C19 genetic test |  |  | <0.0001 |
| 0 LOF alleles (NM, RM, UM) | 9049 (52.5%) | 91 (2.0%) |  |
| 1 LOF allele (IM) | 8193 (47.5%) | 1698 (38.0%) |  |
| 2 LOF alleles (PM) | 0 (0.0%) | 2674 (59.9%) |  |

CKD, Chronic kidney disease; LOF, loss-of-function; NM, CYP2C19 normal metabolizer; PM, CYP2C19 poor metabolizer; RM, CYP2C19 rapid metabolizer; UM, CYP2C19 ultrarapid metabolizer;

^a^ Defined as an estimated glomerular filtration rate of <60 mL/min/1.73 m2 .

^b^ *P* value for comparison of ≥1 LOF allele vs 0 LOF alleles between groups.

**Supplementary Table 3. Distribution of Antiplatelet Therapy Regimens during the 1-Year Follow-Up Period in Clopidogrel and Ticagrelor Groups**

|  | Clopidogrel  (N=15436) | Ticagrelor  (N=6269) |
| --- | --- | --- |
| Antiplatelet therapy during 1 year |  |  |
| Dual antiplatelet therapy | 14077 (91.8%) | 5798 (93.5%) |
| Any P2Y12 inhibitors monotherapy | 910 (5.9%) | 301 (4.9%) |
| Aspirin monotherapy | 184 (1.2%) | 55 (0.9%) |
| None | 1160 (1.0%) | 47 (0.8%) |
| P2Y12 inhibitor switching | 537 (3.5%) | 339 (5.5%) |

**Supplementary Table 4. Baseline and Procedural Characteristics** **Based on P2Y12 Treatment and ABCD-GENE Score before Propensity Score Matching**

|  |  | ABCD-GENE score <10 (N=17242) | | | |  | ABCD-GENE score ≥10 (N=4463) | | | |
| --- | --- | --- | --- | --- | --- | --- | --- | --- | --- | --- |
|  | Clopidogrel (N=12375) | | Ticagrelor (N=4867) | Standardized mean difference | P value | Clopidogrel (N=3061) | | Ticagrelor (N=1402) | Standardized mean difference | P value |
| Age | 61.5±9.5 | | 57.7±9.3 | -0.4006 | <0.0001 | 66.5±11.9 | | 57.3±10.6 | -0.8136 | <0.0001 |
| Male | 8723 (70.5%) | | 3943 (81.0%) | 0.2475 | <0.0001 | 1976 (64.6%) | | 1098 (78.3%) | 0.3083 | <0.0001 |
| Body mass index, kg/m^2 a^ | 24.9±3.0 | | 25.2±3.0 | 0.12242 | <0.0001 | 25.8±4.4 | | 26.9±4.3 | 0.2536 | <0.0001 |
| Medical history |  | |  |  |  |  | |  |  |  |
| Hypertension | 7698 (62.2%) | | 2820 (58.0%) | -0.0872 | <0.0001 | 2103 (68.7%) | | 880 (62.9%) | -0.1239 | 0.0001 |
| Diabetes | 3925 (31.7%) | | 1498 (30.8%) | -0.0203 | 0.2323 | 1167 (38.1%) | | 485 (34.6%) | -0.0735 | 0.0233 |
| Previous MI | 1644 (13.3%) | | 713 (14.7%) | 0.0398 | 0.0179 | 451 (14.8%) | | 203 (14.5%) | -0.008 | 0.8045 |
| Previous stroke | 1767 (14.3%) | | 438 (9.0%) | -0.1651 | <0.0001 | 486 (15.9%) | | 167 (11.9%) | -0.1146 | 0.0005 |
| Previous PCI | 2291 (18.5%) | | 947 (19.5%) | 0.0239 | 0.1562 | 624 (20.4%) | | 270 (19.3%) | -0.0289 | 0.3715 |
| Smoking |  | |  | 0.1734 | <0.0001 |  | |  | 0.2608 | <0.0001 |
| Never | 5843 (47.3%) | | 1884 (38.8%) |  |  | 1673 (54.7%) | | 588 (42.0%) |  |  |
| Active | 4928 (39.9%) | | 2288 (47.1%) |  |  | 1024 (33.5%) | | 623 (44.5%) |  |  |
| Former | 1589 (12.9%) | | 683 (14.1%) |  |  | 360 (11.8%) | | 189 (13.5%) |  |  |
| Presentation |  | |  | 0.21377 | <0.0001 |  | |  | 0.0944 | 0.0134 |
| UA | 8522 (68.9%) | | 2864 (58.8%) |  |  | 1948 (63.6%) | | 828 (59.1%) |  |  |
| NSTEMI | 1809 (14.6%) | | 871 (17.9%) |  |  | 514 (16.8%) | | 262 (18.7%) |  |  |
| STEMI | 2044 (16.5%) | | 1132 (23.3%) |  |  | 599 (19.6%) | | 312 (22.3%) |  |  |
| eGFR, ml/min/1.73m^2 b^ | 93.2±24.5 | | 96.5±23.1 | 0.1375 | <0.0001 | 82.0±29.4 | | 92.8±25.7 | 0.3915 | <0.0001 |
| Anemia ^c^ | 4462 (36.2%) | | 1596 (32.8%) | -0.0704 | <0.0001 | 1424 (46.6%) | | 491 (35.1%) | -0.2347 | <0.0001 |
| LVEF | 58.1±8.6 | | 57.5±8.4 | -0.0604 | 0.0017 | 56.4±9.1 | | 57.1±8.7 | 0.0812 | 0.0265 |
| Procedure information |  | |  |  |  |  | |  |  |  |
| Transradial access | 11574 (93.8%) | | 4579 (94.2%) | 0.0177 | 0.2986 | 2833 (92.6%) | | 1329 (95.0%) | 0.099 | 0.003 |
| Coronary arteries treated |  | |  |  |  |  | |  |  |  |
| LM | 575 (4.6%) | | 327 (6.7%) | 0.0896 | <0.0001 | 169 (5.5%) | | 67 (4.8%) | -0.0336 | 0.3038 |
| LAD | 6661 (53.8%) | | 2637 (54.2%) | 0.0071 | 0.6738 | 1597 (52.2%) | | 766 (54.6%) | 0.0494 | 0.1259 |
| LCX | 3234 (26.1%) | | 1288 (26.5%) | 0.0075 | 0.6569 | 814 (26.6%) | | 399 (28.5%) | 0.0418 | 0.1932 |
| RCA | 4902 (39.6%) | | 1771 (36.4%) | -0.0665 | <0.0001 | 1237 (40.4%) | | 510 (36.4%) | -0.083 | 0.0104 |
| Number of stents | 1.6±1.0 | | 1.7±1.0 | 0.1344 | <0.0001 | 1.6±1.0 | | 1.6±1.0 | 0.0613 | 0.0574 |
| Total length of stents, mm | 41.1±28.1 | | 45.4±28.6 | 0.1533 | <0.0001 | 41.4±28.0 | | 43.9±28.3 | 0.0881 | 0.0062 |
| Average stent diameters, mm | 2.8±0.9 | | 2.9±0.8 | 0.1495 | <0.0001 | 2.7±0.9 | | 2.8±0.8 | 0.1161 | 0.0003 |
| Medical treatment at discharge |  | |  |  |  |  | |  |  |  |
| Aspirin | 11684 (94.4%) | | 4848 (99.6%) | 0.3087 | <0.0001 | 2829 (92.4%) | | 1396 (99.6%) |  | <0.0001 |
| Statins | 11072 (89.5%) | | 4543 (93.3%) | 0.1385 | <.0001 | 2699 (88.2%) | | 1324 (94.4%) |  | <0.0001 |
| ACEI/ARB | 5744 (46.4%) | | 2679 (55.0%) | 0.1732 | <0.0001 | 1481 (48.4%) | | 796 (56.8%) |  | <0.0001 |
| βblockers | 6579 (53.2%) | | 3131 (64.3%) | 0.2283 | <0.0001 | 1644 (53.7%) | | 920 (65.6%) |  | <0.0001 |
| PPI | 3933 (31.8%) | | 1632 (33.5%) | 0.0373 | 0.0269 | 952 (31.1%) | | 434 (31.0%) |  | 0.9225 |
|  |  | |  | -0.4006 |  |  | |  |  |  |

Abbreviations: ACEI, angiotensin-converting enzyme inhibitor; ARB, angiotensin II receptor blocker; CABG, coronary artery bypass grafting; eGFR, estimated glomerular filtration rate; LVEF, left ventricular ejection fraction; LM, left main coronary artery; LAD, left anterior decending branch; LCX, left circumflex branch; MI, myocardial infarction; NSTEMI, Non-ST -segment-elevation myocardial infarction; PCI, percutaneous coronary intervention; PPIs, proton pump inhibitors; RCA, right coronary artery; STEMI, ST-segment-elevation myocardial infarction; UA, unstable angina.

^a^ Calculated as weight in kilograms divided by height in meters squared.

^b^ Calculated as milliliters per minute per 1.73 square meters.

^c^ Anemia was defined as hemoglobin (less than 130 g/L for male patients and less than 120 g/L for female patients.

**Supplementary Table 5. Clinical Outcomes Based on P2Y12 Treatment and ABCD-GENE Score before Propensity Score Matching before Propensity Score Matching**

|  | ABCD-GENE score <10 (N=17242) | | | | ABCD-GENE score ≥10 (N=4463) | | | |
| --- | --- | --- | --- | --- | --- | --- | --- | --- |
|  | Clopidogrel (N=12375) | Ticagrelor (N=4867) | HR (95%CI) | P value | Clopidogrel (N=3061) | Ticagrelor (N=1402) | HR (95%CI) | P value |
| Primary outcome | 441 (3.6%) | 175 (3.6%) | 1.01 (0.85-1.20) | 0.9332 | 163 (5.3%) | 67 (4.8%) | 0.89 (0.67-1.18) | 0.4287 |
| Ischemic events | 300 (2.4%) | 80 (1.6%) | 0.67 (0.53-0.86) | 0.0017 | 117 (3.8%) | 31 (2.2%) | 0.57 (0.38-0.85) | 0.0056 |
| Cardiac death | 141 (1.1%) | 38 (0.8%) | 0.68 (0.48-0.98) | 0.0377 | 76 (2.5%) | 13 (0.9%) | 0.37 (0.21-0.67) | 0.0009 |
| MI | 71 (0.6%) | 26 (0.5%) | 0.93 (0.59-1.45) | 0.7401 | 22 (0.7%) | 9 (0.6%) | 0.88 (0.41-1.92) | 0.7523 |
| Stroke | 111 (0.9%) | 23 (0.5%) | 0.52 (0.33-0.82) | 0.0047 | 33 (1.1%) | 10 (0.7%) | 0.65 (0.32-1.32) | 0.2354 |
| All-cause death | 193 (1.6%) | 44 (0.9%) | 0.58 (0.42-0.80) | 0.001 | 106 (3.5%) | 17 (1.2%) | 0.35 (0.21-0.58) | <0.0001 |
| BARC type 2,3 or 5 bleeding | 707 (5.7%) | 449 (9.2%) | 1.65 (1.47-1.86) | <0.0001 | 178 (5.8%) | 145 (10.3%) | 1.81 (1.45-2.25) | <0.0001 |
| BARC type 3 or 5 bleeding | 162 (1.3%) | 97 (2.0%) | 1.52 (1.18-1.96) | 0.001 | 53 (1.7%) | 36 (2.6%) | 1.47 (0.96-2.25) | 0.0727 |

Abbreviation: BARC, Bleeding Academic Research Consortium; CI, confidence interval; HR = hazard ratio; MI, myocardial infarction.

The primary outcome was deﬁned as the composite of cardiac death, MI, stroke, or BARC types 3 or 5 bleeding.
